# Subsequent Pregnancies After Conservative Placenta Accreta Management: Recurrent Accreta and Preserved Fertility, a Systematic Review and Meta-Analysis

**DOI:** 10.3390/jcm15051684

**Published:** 2026-02-24

**Authors:** Shmuel Somer, Doron Kabiri, Lauren H. Yaeger, Shmuel Herzberg, Yossef Ezra, Aharon Tevet, Joshua I. Rosenbloom

**Affiliations:** 1 Department of Obstetrics and Gynecology, Sheba Medical Center, Ramat Gan 52621, Israel; 2 Department of Obstetrics and Gynecology, Hadassah Medical Organization, Faculty of Medicine, Hebrew University of Jerusalem, Jerusalem 91120, Israel; doronkabiri@gmail.com (D.K.); shmuelh@hadassah.org.il (S.H.); ezrayossi69@gmail.com (Y.E.); aharont@hadassah.org.il (A.T.); joshuar@hadassah.org.il (J.I.R.); 3 Bernard Becker Medical Library, Washington University School of Medicine, St. Louis, MO 63110, USA

**Keywords:** placenta accreta spectrum, conservative management, recurrent placenta accreta, fertility preservation, subsequent pregnancy outcomes, systematic review and meta-analysis

## Abstract

**Background:** Placenta accreta spectrum (PAS) is a serious obstetric condition characterized by abnormal placental adherence to the uterus that can lead to major maternal morbidity. While hysterectomy has traditionally been the standard management, uterus-preserving approaches are increasingly used to preserve fertility. The risk of recurrent PAS in subsequent pregnancies and the overall fertility outcomes following conservative management remain unclear. **Objective:** We aimed to estimate the recurrence risk of PAS in subsequent pregnancies after conservative management and to assess fertility outcomes, including pregnancy and live-birth rates. **Methods:** This systematic review and meta-analysis followed PRISMA guidelines. A comprehensive literature search was performed across multiple databases to identify studies reporting subsequent pregnancies after conservative PAS management. Data extraction and quality assessment were independently conducted. Pooled recurrence and pregnancy success rates were calculated using random-effects meta-analysis. **Results:** Eleven studies met the inclusion criteria, involving 2642 patients who underwent conservative PAS management. The pooled recurrence risk of PAS in subsequent pregnancies was 20.9% (95% CI: 12.2–29.6). Successful pregnancy rates following conservative treatment were 69.7% (95% CI: 49.9–89.5). **Conclusions:** While conservative PAS management poses a risk of recurrence, it remains a viable fertility-preserving option, with high subsequent pregnancy success rates. These findings support informed clinical decision-making, though further prospective studies are needed to optimize management strategies and patient outcomes.

## 1. Introduction

Placenta accreta spectrum (PAS) refers to abnormal placental adherence or invasion to the uterus, in which attempting detachment can result in life-threatening hemorrhage [1]. PAS includes placenta accreta, increta, and percreta, defined by abnormal adherence to or invasion of the myometrium, rather than being confined within the decidua [2]. These conditions pose major risks to maternal health and require timely diagnosis and management [3]. Complications include maternal hemorrhage, infection, sepsis, disseminated intravascular coagulation, multiorgan failure, and maternal mortality [4,5]. Historically, cesarean hysterectomy has been the standard approach to mitigate the associated risks [4,6].

Over the past four decades, there has been an approximate five- to tenfold increase in the incidence of PAS in developed countries [7], largely paralleling rising rates of cesarean delivery and other uterine surgery [2]. Placenta previa overlying a uterine scar is the strongest risk factor, with additional contributions from advanced maternal age, multiparity, and assisted reproductive technologies [8]. As these risk factors become more common globally, PAS has emerged as a growing cause of obstetric morbidity, emphasizing the importance of optimizing both peripartum management and long-term reproductive outcomes [8,9]. Parallel advances in conservative and fertility-preserving surgical techniques have provided alternatives to hysterectomy. Multiple conservative, uterus-preserving strategies have been described, including leaving the placenta in situ, uterine artery embolization, internal iliac artery ligation, balloon tamponade, uterine compression sutures such as the B-Lynch technique, and, more recently, modified one-step conservative uterine surgery (MOSCUS) [10]. Conservative management is increasingly considered in carefully selected patients who desire future fertility [10,11]. For instance, the modified one-step conservative uterine surgery (MOSCUS) method demonstrated reduced operative time, estimated blood loss, and red blood cell transfusion requirements [12]. Such approaches enable uterine preservation and the potential for future pregnancies [7].

Given this trend toward uterus-preserving management, evaluating the risk of recurrent PAS in subsequent pregnancies is essential [13]. Understanding recurrence risk and fertility potential is crucial for informed clinical decision-making. Recent observational studies have examined outcomes of subsequent pregnancies after conservative management [14]; however, specific recurrence rates of PAS disorders remain incompletely defined. Recurrence rates of PAS in subsequent pregnancies vary widely across individual studies. Variability in diagnostic definitions, heterogeneity in conservative methods, and inconsistent reporting of subsequent pregnancy outcomes contribute to uncertainty [14,15]. A comprehensive synthesis of recurrence risk and fertility outcomes is therefore needed to guide counseling and clinical decision-making. Clarifying these risks will better equip healthcare professionals to provide targeted counseling and management for patients affected by PAS.

This systematic review and meta-analysis primarily aimed to determine the recurrence risk of PAS in subsequent pregnancies following uterine conservation, and secondarily, to evaluate fertility outcomes, including pregnancy and live-birth rates.

## 2. Materials and Methods

The following systematic review and meta-analysis was carried out in alignment with the Preferred Reporting Items for Systematic Reviews and Meta-analyses (PRISMA) guidelines [13]. No protocol was registered for this systematic review. The review methods were established in advance and conducted according to PRISMA 2020 guidelines (see Supplementary Material: PRISMA checklist).

### 2.1. Search Strategy

A medical librarian (LHY) conducted a comprehensive search for studies addressing PAS and conservative, uterine-sparing management. The search combined keywords and controlled vocabulary across Embase.com 1947-, Ovid Medline 1946-, Scopus 1823-, Cochrane Central Register of Controlled Trials (CENTRAL), The Cochrane Database of Systematic Reviews (CDSR), and Clinicaltrials.gov 1997-. All search strategies were completed on 18 July 2022 with no language or date limits applied. Search results were exported to Endnote and Covidence for deduplication and screening. Fully reproducible search strategies for each database can be found in the Appendix A. Originally, studies published after 18 July 2022 were outside the predefined search period. However, all searches were updated from database inception to 22 December 2025 and de-duplicated against the original results using Covidence.org to find 429 new results.

### 2.2. Eligibility Criteria

Studies were eligible if they included women with a history of PAS managed conservatively who had at least one subsequent reported pregnancy. Eligible studies were required to report at least one outcome related to recurrent PAS. Studies that did not report subsequent pregnancies were excluded. Case reports and single-patient descriptions were excluded. There were no limitations on the language of publication.

To ensure methodological consistency across studies, eligibility criteria were further defined according to the Population, Intervention, Comparator, Outcome (PICO) framework. The population included women with a diagnosis of placenta accreta spectrum who underwent conservative, uterus-preserving management. Eligible interventions encompassed any conservative strategy, including leaving the placenta in situ, uterine artery embolization, and other uterine-sparing surgical techniques. A comparator group was not required, and both comparative and non-comparative cohort studies were eligible. Studies were required to report outcomes related to subsequent pregnancy, live birth, or recurrent PAS. We included cohort studies, case–control studies, and case series with more than ten participants. Exclusion criteria included case reports, small case series with fewer than ten cases, review articles, conference abstracts without available full text, studies that did not report subsequent pregnancies, and studies with overlapping patient populations.

### 2.3. Study Selection

A two-stage screening process was used, consisting of initial title and abstract screening followed by full-text review for all potentially eligible studies. Two reviewers (SS and JIR) screened all records independently. Discrepancies at any stage were resolved through discussion and consensus. Covidence (Veritas Health Innovation, Melbourne, Australia; https://www.covidence.org), accessed January 2026, was used to manage screening, de-duplication, and reviewer agreement. The PRISMA flow diagram outlines the number of records identified, screened, excluded, and included at each stage. Originally, studies published after 18 July 2022 were outside the predefined search period. However, all searches were updated from database inception to 22 December 2025 and de-duplicated against the original results using Covidence.org to find 429 new results.

### 2.4. Data Extraction

Data extraction was performed independently by two reviewers (SS and JIR) using a standardized data extraction form developed prior to analysis. Extracted variables included study characteristics (country, years of data collection, design, and sample size), diagnostic criteria for PAS, type of conservative management performed, number of subsequent pregnancies, number of live births, number of recurrent PAS cases, and duration of follow-up. When information was unclear or incomplete, the data available in the published manuscripts were used without imputation, and study authors were not contacted.

### 2.5. Quality Appraisal

Risk of bias was assessed for all included studies using the Newcastle–Ottawa Scale (NOS). This tool was selected because it allows structured assessment across three domains: selection of study participants, comparability of groups, and adequacy of outcome measurement. Two reviewers (SS and JIR) independently evaluated each study according to the NOS criteria. Any discrepancies in scoring were resolved through discussion until consensus was reached. Overall, methodological quality ranged from poor to good across the included studies. The detailed NOS ratings for each study are presented in Table 1.

### 2.6. Statistical Analysis

Descriptive and outcome data were extracted from all included studies. Meta-analyses were performed using Stata version 14 (StataCorp, College Station, TX, USA). Pooled proportions and corresponding 95% confidence intervals were estimated using DerSimonian–Laird random-effects models, which account for expected clinical and methodological heterogeneity across studies. Statistical heterogeneity was assessed using standard measures of between-study variability as implemented by the software. Sensitivity analyses were considered to explore the influence of individual studies, but the limited number of eligible studies precluded formal testing. Assessment of publication bias was not performed because fewer than ten studies were available for pooling, consistent with established methodological recommendations. Results are presented in tables and forest plots.

## 3. Results

### 3.1. Search Results

**Figure 1:**
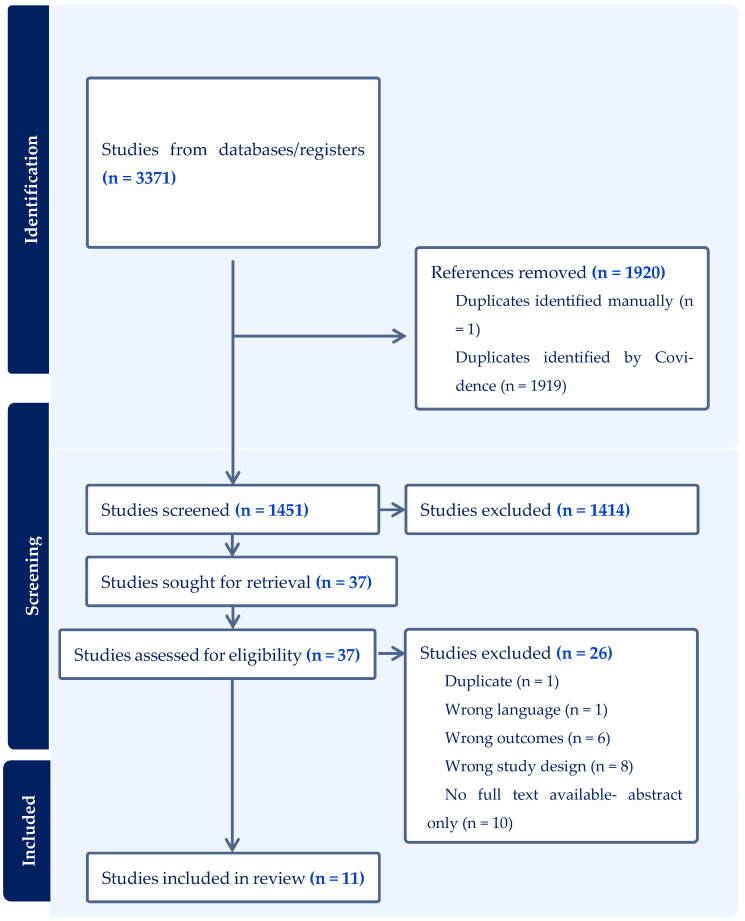
PRISMA Diagram.

Of the 3371 studies retrieved from the databases and registers, 1920 were removed as duplicates, and 1451 studies were subsequently screened. Of 1451 unique records screened by two independent reviewers, 1414 were excluded as irrelevant, leaving 37 full-text articles for eligibility assessment. Of these, eleven studies met the inclusion criteria and were included in the final analysis (Table 2) [15,16,17]. Reasons for exclusion included insufficient data on subsequent pregnancies, overlapping patient populations, literature reviews, and case reports (Appendix B). Originally, studies published after 18 July 2022 were outside the predefined search period. However, all searches were updated from database inception to 22 December 2025 and de-duplicated against the original results using Covidence.org to find 429 new results. The PRISMA flow diagram summarizes study selection at each stage based on the latest search updates (Figure 1).

### 3.2. Study Characteristics

Eleven studies met the inclusion criteria and were included in the analysis. The studies were conducted between 1988 and 2025. The studies were conducted in Israel, the United States, Australia, France, Egypt, Germany, Pakistan, and China. Sample sizes ranged from 6 patients to 1222 women, for a total of 2642 cases managed conservatively for PAS. Reported conservative techniques included leaving the placenta in situ, bilateral uterine artery embolization, Bakri balloon catheter placement, placental bed sutures, B-Lynch sutures, chitosan tamponade insertion, one-step conservative surgery, and other uterine-preserving techniques. In subsequent pregnancies, the number of live births per study ranged from 2 to 565, totaling 956 across all studies. Overall, study quality was poor to good based on the Newcastle–Ottawa Scale (Table 1).

### 3.3. Outcomes

**Figure 2:**
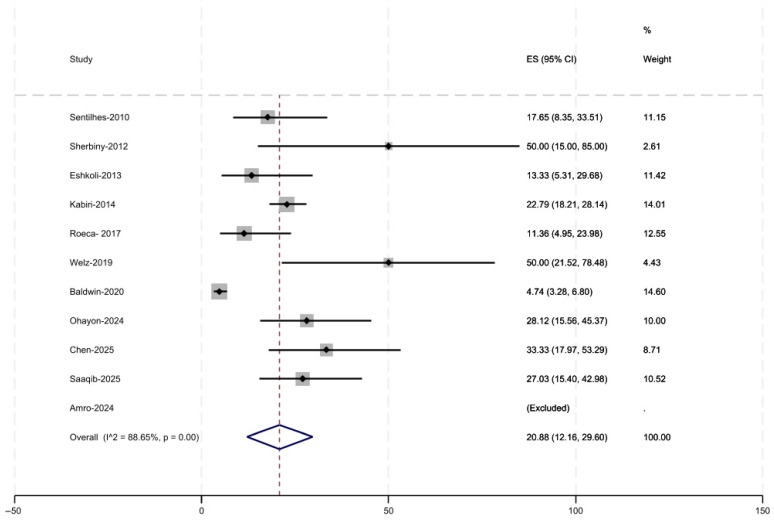
Rate of Recurrent Accreta [15,16,17,18,19,20,21,22,23,24,25].

**Figure 3:**
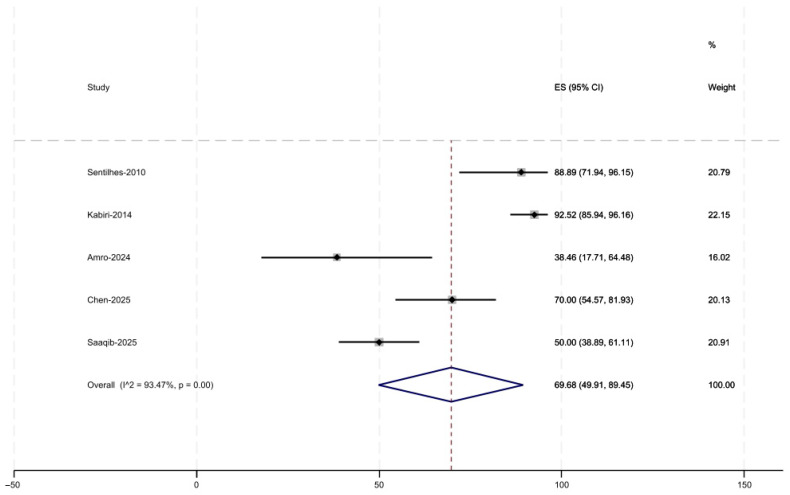
Rate of Successful Pregnancies [16,17,20,23,24].

Of the eleven included studies, ten reported the primary outcome of recurrent PAS. Reported recurrence rates of PAS in subsequent pregnancies ranged from 4.7% to 50% (Figure 2). The pooled estimate was 20.9% (95% CI, 12.2–29.6). Five studies reported the secondary outcome of successful pregnancies, with rates ranging from 38.5% to 92.5% (Figure 3). The pooled success rate was 69.7% (95% CI, 49.9–89.5). The variation in results likely reflects differences in patient selection, definitions of PAS, and follow-up duration across studies.

## 4. Discussion

This systematic review and meta-analysis evaluated the recurrence of placenta accreta spectrum and subsequent fertility outcomes following conservative management. In this systematic review and meta-analysis, the pooled rate of recurrent PAS after conservative management was 20.1% (95% CI, 12.2–29.6), and the rate of successful subsequent pregnancies was 69.7% (95% CI, 49.9–89.5). This pooled estimate aligns with the range reported across prior observational studies examining subsequent pregnancy after conservative management for PAS. This suggests that although recurrence is not rare, occurring in approximately one in six women, conservative management still allows a high rate of successful subsequent pregnancies. Since our search date, additional cohorts and systematic reviews have reported subsequent pregnancies after conservative management, with recurrent PAS ranging roughly from 12% to 33% [14,18]. These findings indicate that conservative management remains a viable fertility-preserving option for appropriately selected women, despite the inherent risk of recurrence.

Clinically, these findings provide valuable insight into the management of PAS in women seeking to preserve their fertility. According to the American College of Obstetricians and Gynecologists (ACOG) guidelines, conservative management of PAS is considered when fertility preservation is desired [2]. Our findings align with these guidelines, emphasizing that although there is a notable risk of recurrent PAS, the likelihood of subsequent successful pregnancies is high. For example, Chen et al. reported a 33.3% recurrence rate in a single-center ambispective cohort study [18]. Similarly, Javinani et al. reported a recurrence rate of 11.8% (95% CI, 11.7–60.3) [14]. This difference likely reflects variations in inclusion criteria, as our analysis incorporated the studies by Kabiri, Sherbiny, Welz, and Eshkoli, which were excluded from theirs. Nguyen et al. evaluated neonatal outcomes, comparing PAS cases managed by emergency cesarean delivery versus planned hysterectomy or the MOSCUS technique [19]. They found that emergency cesarean delivery was associated with higher neonatal morbidity compared with either planned hysterectomy or MOSCUS-based conservative management [19]. Contemporary reports on conservative techniques, notably MOSCUS, also demonstrate improved perioperative outcomes at the index delivery compared with cesarean hysterectomy, which may partly explain the growing adoption of uterus-preserving strategies [19].

Strengths of this study include a comprehensive search strategy developed with an experienced medical librarian, independent screening by two reviewers, and adherence to PRISMA guidelines. These methods ensured a thorough and transparent review of the relevant literature. Additionally, the data extraction was updated to 22 December 2025, thus ensuring the most up-to-date literature in the analysis.

Limitations include heterogeneity in conservative techniques, which may reduce the generalizability of the findings. Additionally, the diagnosis of PAS was not standardized across studies. The secondary outcome, subsequent successful pregnancy, was based on limited data from only five studies, restricting the strength of clinically meaningful conclusions.

Future research should focus on identifying predictors of favorable fertility outcomes and strategies to reduce recurrence risk, including the role of interpregnancy interval and other modifiable risk factors. Prospective registries or multi-center cohort studies are needed to clarify long-term reproductive outcomes in the modern era.

## 5. Conclusions

Conservative management of PAS is associated with a measurable risk of recurrence in subsequent pregnancies, while many women who desire future fertility can conceive successfully. These findings highlight the importance of individualized counseling and careful planning for future pregnancies in women managed conservatively. Additional prospective studies with standardized definitions and long-term follow-up are needed to better characterize reproductive outcomes after conservative treatment.

## Figures and Tables

**Table 1:** Risk of Bias Assessment—Newcastle–Ottawa Scale [15,16,17,18,19,20,21,22,23,24,25].

Author and Year of Publication	Selection	Comparability	Outcome	Median Duration of Follow-Up	Quality
Baldwin-2020 [15]	4/4	2/2	3/3	5 years	Good
Kabiri-2014 [16]	4/4	1/2	3/3	Follow up was 10 years after the study	Good
Sentilhes-2010 [20]	3/4	0/2	2/3	2–172 months	Poor
Sherbiny-2012 [21]	3/4	0/2	2/3	2 months-2 years	Poor
Welz-2019 [22]	3/4	0/2	1/3	6 months-14 years	Poor
Roeca-2017 [19]	3/4	0/2	3/3	Unspecified	Poor
Eshkoli-2013 [18]	3/4	1/2	2/3	Unspecified	Good
Amro-2025 [23]	4/4	2/2	0/3	Unspecified	Fair
Chen-2025 [24]	4/4	1/2	3/3	Unspecified	Good
Ohayon-2025 [25]	4/4	2/2	2/3	Unspecified	Good
Saaqib-2025 [17]	4/4	1/2	3/3	5 years	Good

**Table 2:** Characteristics of included studies evaluating conservative management of placenta accreta spectrum (PAS), including reproductive outcomes and diagnostic criteria [15,16,17,18,19,20,21,22,23,24,25].

Author and Year	Years and Location of Study	Type of Conservative Treatment(s) Used	N of Patients in the Study	Attempted Pregnancies	Pregnancy Achieved	Live Births	Recurrent PAS	Diagnosis of PAS
Baldwin-2020 [15]	2003–2016 Australia	-	1222	-	-	565	27	International Classification of Diseases, 10th Revision, Australian Modification diagnosis code for morbidly adherent placenta.
Eshkoli-2013 [18]	1988–2011 Israel	-	139	-	30	-	4	Clinical diagnosis. Only included cesarian deliveries and based on difficulties of the physician removing the placenta during the operation.
Kabiri-2014 [16]	1990–2000, Pregnancies until 2010 Israel	-	134	107	99	280	62	Patients who met one or more of the following criteria: 1. Impossibility of, or incomplete, manual removal of the placenta with evidence of placental retention, despite active management of the third stage of labor. 2. Sonographic evidence of retained placental fragments requiring removal after vaginal delivery. 3. Heavy bleeding from the implantation site after placental removal during cesarean delivery with excision of part of the uterine wall and the attached placenta or oversewing of the bleeding defects. 4. Histologic confirmation of placenta accreta.
Roeca-2017 [19]	2007–2015	-	339	-	-	39	21	Confirmed by pathologic diagnosis. Pathology sent based on provider’s discretion
Sentilhes-2010 [20]	1993–2007 United States	Leaving placenta in situ	131	27	24	2	6	The clinical criteria for diagnosis were one of the following: 1. Manual removal of the placenta was partially or totally impossible and there was no cleavage plane between part or all the placenta and the uterus. 2. The prenatal diagnosis of placenta accreta was confirmed by the failure of a gentle attempt at removal during the third stage of labor.
Sherbiny-2012 [21]	Egypt	Uterine artery bilateral double ligation	10	-	-	4	2	Diagnosed by ultrasound findings.
Welz-2019 [22]	2003–2017 Germany	Bilateral uterine artery embolization, Bakri catheter, placental bed suture, B-Lynch suture, and Chitosan tamponade insertion	39 managed conservatively. 24 lost to follow up.	-	-	8	4	Prenatal included color Doppler ultrasound. Some patients underwent subsequent MR or cystoscopy depending on area of suspected invasion. During labor it was defined by clinical criteria based on severity of hemorrhage and depth of invasion.
Amro-2025 [23]	2015–2024 United States	Leaving placenta in situ	13 Successful Uterine Preservation	-	5	-	0	Antenatal ultrasound suspicion of PAS with intraoperative confirmation
Chen-2025 [24]	2013–2021 China	Extirpative placental removal, leaving placenta in situ, or one-step conservative surgery	178 conservatively managed	40	43	23	8	Prenatal ultrasound/MRI with intraoperative or postpartum clinical findings, supported by pathology.
Ohayon-2025 [25]	2011–2022 Israel	One-step uterine-preserving surgery with placental removal and uterine reconstruction.	287	-	-	32	9	Prenatal ultrasound with clinical/intraoperative confirmation using FIGO PAS classification.
Saaqib-2025 [17]	2017–2024 Pakistan	Leaving placenta in situ	150	74	37	-	10	Prenatal ultrasound ± MRI with intraoperative clinical confirmation; pathology when tissue available.

## Data Availability

The dataset used and analyzed during the current study is available from the corresponding author upon reasonable request.

## Supplementary Materials

The following supporting information can be downloaded at: https://www.mdpi.com/article/10.3390/jcm15051684/s1, Supplementary Material: PRISMA checklist.

## Abbreviations

The following abbreviations are used in this manuscript:

PAS	Placenta Accreta Spectrum
ACOG	American College of Gynecology
CI	Confidence Interval

## Appendix A

Full Search Strategies
*Embase*
*Date Searched: 18 July 2022*
*Applied Database Supplied Limits: none*
*Number of Results: 923*
*Search updated:* 22 December 2025
*Results:* 1274
*Full Search Strategy:*
*(‘placenta accreta’/exp OR ‘placenta accreta spectrum’/exp OR ‘placenta accreta spectrum disorder’/exp OR (placenta* NEAR/3 (accrete OR percreta OR increta)):ti,ab,kw OR (‘abnormal placental attachment’ OR ‘placental attachment disorder’):ti,ab,kw) AND (‘conservative treatment’/exp OR ‘organ preservation’/exp OR ‘organ sparing surgery’/exp OR ((conservative OR nonoperative OR ‘non operative’ OR ‘non-operative’ OR nonsurgical) NEAR/3 (treatment* OR management OR therap*)):ti,ab,kw OR ((uterine OR uterus OR organ) NEAR/3 (conserve* OR sparing OR spare* OR preserv*)):ti,ab,kw OR (remov* NEAR/3 placenta):ti,ab,kw)*
Ovid Medline
*Date Searched:* 18 July 2022
*Applied Database Supplied Limits: none*
*Number of Results: 452*
*Search updated:* 22 December 2025
*Results: 559*
*Full Search Strategy:*
*(exp Placenta Accreta/OR (placenta* ADJ3 (accrete OR percreta OR increta)).ti,ab,kf. OR (abnormal placental attachment OR placental attachment disorder).ti,ab,kf.) AND (exp Conservative Treatment/OR exp Organ Preservation/OR exp Organ Sparing Treatments/OR ((conservative OR nonoperative OR non operative OR non-operative OR nonsurgical) ADJ3 (treatment* OR management OR therap*)).ti,ab,kf. OR ((uterine OR uterus OR organ) ADJ3 (conserve* OR sparing OR spare* OR preserv*)).ti,ab,kf. OR (remov* ADJ3 placenta).ti,ab,kf.)*
***Scopus***
*Date Searched: 18 July 2022*
*Applied Database Supplied Limits: none*
*Number of Results: 353*
*Search updated: 22 December 2025*
*Results: 437*
*Full Search Strategy:*
*((TITLE-ABS-KEY(placenta* W/3 (accrete OR percreta OR increta))) OR (TITLE-ABS-KEY(“abnormal placental attachment” OR “placental attachment disorder”))) AND ((TITLE-ABS-KEY((conservative OR nonoperative OR “non operative” OR “non-operative” OR nonsurgical) W/3 (treatment* OR management OR therap*))) OR (TITLE-ABS-KEY((uterine OR uterus OR organ) W/3 (conserve* OR sparing OR spare* OR preserv*))) OR (TITLE-ABS-KEY(remov* W/3 placenta)))*
***The Cochrane Library***
*Date Searched: 18 July 2022*
*Applied Database Supplied Limits: none*
*Number of Results*
*CENTRAL: 14*
*CDSR: 0*
*Search updated: 22 December 2025*
*Results*
*CENTRAL: 23*
*CDSR: 0*
*Full Search Strategy:*
*([mh “Placenta Accreta”] OR (placenta* NEAR/3 (accrete OR percreta OR increta)):ti,ab,kw OR (“abnormal placental attachment” OR “placental attachment disorder”):ti,ab,kw) AND ([mh “Conservative Treatment”] OR [mh “Organ Preservation”] OR [mh “Organ Sparing Treatments”] OR ((conservative OR nonoperative OR “non operative” OR “non operative” OR nonsurgical) NEAR/3 (treatment* OR management OR therap*)):ti,ab,kw OR ((uterine OR uterus OR organ) NEAR/3 (conserve* OR sparing OR spare* OR preserv*)):ti,ab,kw OR (remov* NEAR/3 placenta):ti,ab,kw)*
***ClinicalTrials.gov***
*Date Searched: 18 July 2022*
*Number of Results: 26*
*Search updated: 12/22/2025*
*Results: 54*
*Full Search Strategy:*
*“Placenta Accreta” AND (conservative OR “non-operative”)*

## Appendix B

Reasons For Exclusion
Bretelle 2007 [26]—The same patients were analyzed in the reported Sentilhes study.
Courbière 2003 [27]—This did not include information on recurrent placenta accretas.
Cunningham 2015 [28]—This is a literature review.
Mei 2015 [29]—This is a literature review.
Pineles 2023 [30]—This did not assess for subsequent placenta accreta.
Provansal 2010 [6]—The same patients were analyzed in the reported Sentilhes study.
Verspyck 2005 [31]—Recurrent placenta accretas were not confirmed.
Pineles 2024 [32]—The same patients were analyzed in the reported Amros study.
Hwu 2005 [33]—This is a case report.
Legendre 2012 [34]—This did not assess for subsequent placenta accreta.
